# Genome-wide investigation of the TGF-β superfamily in scallops

**DOI:** 10.1186/s12864-023-09942-w

**Published:** 2024-01-02

**Authors:** Qian Zhang, Jianming Chen, Wei Wang, Jingyu Lin, Jiabao Guo

**Affiliations:** 1 Institute of Oceanography, College of Geography and Oceanography, Minjiang University, Fuzhou, 350108 China; 2 Fujian Key Laboratory on Conservation and Sustainable Utilization of Marine Bioaffiliationersity, Minjiang University, Fuzhou, 350108 China

**Keywords:** Scallop, TGF-β superfamily, Phylogeny, Gene expression, Genome-wide

## Abstract

**Background:**

Transforming growth factor β (TGF-β) superfamily genes can regulate various processes, especially in embryogenesis, adult development, and homeostasis. To understand the evolution and divergence patterns of the TGF-β superfamily in scallops, genome-wide data from the Bay scallop (*Argopecten irradians*), the Zhikong scallop (*Chlamys farreri*) and the Yesso scallop (*Mizuhopecten yessoensis*) were systematically analysed using bioinformatics methods.

**Results:**

Twelve members of the TGF-β superfamily were identified for each scallop. The phylogenetic tree showed that these genes were grouped into 11 clusters, including BMPs, ADMP, NODAL, GDF, activin/inhibin and AMH. The number of exons and the conserved motif showed some differences between different clusters, while genes in the same cluster exhibited high similarity. Selective pressure analysis revealed that the TGF-β superfamily in scallops was evolutionarily conserved. The spatiotemporal expression profiles suggested that different TGF-β members have distinct functions. Several BMP-like and NODAL-like genes were highly expressed in early developmental stages, patterning the embryonic body plan. GDF8/11-like genes showed high expression in striated muscle and smooth muscle, suggesting that these genes may play a critical role in regulating muscle growth. Further analysis revealed a possible duplication of AMH, which played a key role in gonadal growth/maturation in scallops. In addition, this study found that several genes were involved in heat and hypoxia stress in scallops, providing new insights into the function of the TGF-β superfamily.

**Conclusion:**

Characteristics of the TGF-β superfamily in scallops were identified, including sequence structure, phylogenetic relationships, and selection pressure. The expression profiles of these genes in different tissues, at different developmental stages and under different stresses were investigated. Generally, the current study lays a foundation for further study of their pleiotropic biological functions in scallops.

**Supplementary Information:**

The online version contains supplementary material available at 10.1186/s12864-023-09942-w.

## Background

Scallops are bivalve molluscs that belong to the family *Pectinidae* and are widely distributed worldwide. Scallops play a critical role in benthic ecology, and many species are economically important fisheries and aquaculture species, providing high quality protein food for humans [1]. Improvement of growth-related traits is a major focus of scallop breeding. Investigating the genetic regulation of scallop growth could benefit scallop breeding. The transforming growth factor β (TGF-β) superfamily plays critical roles in cell proliferation, differentiation, adhesion, migration, and apoptosis [2–4], and is therefore a plausible candidate growth regulator in scallops.

The TGF-β superfamily is an evolutionarily conserved family of secreted polypeptide factors that has undergone minor changes in invertebrates and vertebrates [3]. A common characteristic of this family of proteins is the presence of 6–9 and usually 7 conserved cysteine residues [4]. Six of the cysteine residues form intramolecular disulfide bonds, and the seventh cysteine forms an intermolecular disulfide bond responsible for the covalent linkage of two subunits of the dimeric protein [5]. The TGF-β superfamily consists of a large group of cell regulatory proteins, such as TGF-βs (TGF-β1/2/3), Nodal, activin/inhibin, left-right determination factor (LEFTY), bone morphogenetic protein (BMP), growth and differentiation factor (GDF), anti-dorsalising morphogenetic protein (ADMP) and other superfamily genes [6]. The members are diverse and exhibit tissue-specific and developmental stage-dependent biological effects.

The TGF-β superfamily plays crucial roles in the development and homeostasis of several vital processes, including embryo differentiation, neurogenesis, cell cycle, apoptosis, mesoderm and endoderm induction, and left-right axis determination [4, 7]. In addition, the TGF-β superfamily plays a key role in muscle growth and development [8]. For example, GDF8, also known as myostatin (MSTN), is a conservative regulator of muscle growth and has become one of the most important target genes for genetic improvement in aquatic animals [9]. In scallops, the analysis of TGF-β superfamily genes has mainly focused on GDF8 [10–12]. However, few studies have suggested other functions of TGF-β members in scallops. In addition, several members of the TGF-β superfamily have also been identified as sex determination/differentiation genes [6]. For example, GDFs and BMPs are involved in both male and female germ cell growth and differentiation in scallops [13]. OgTGF-β has been implicated as an activator of germ cell development in oysters, and inhibition of ogTGF-β function tends to reduce the gonadal area [14, 15]. BMP, GDF, gonadal soma-derived factor (GSDF), activin and anti-Müllerian hormone (AMH) have also been identified as master sex-determining genes in some fish species [4, 6, 16–18]. Additionally, the TGF-β superfamily is an essential immunomodulatory molecular switch and is therefore important for the homeostatic maintenance of the immune system [19, 20].

Analyses of the TGF-β superfamily in scallops have thus far been limited to single species [13]. Expression profiles of most of the TGF-β superfamily genes in different tissues and developmental stages are still lacking. To date, several questions about the TGF-β superfamily in the scallop remain unanswered. For example, how many types of the TGF-β superfamily are present in scallops? How many TGF-β superfamily genes are present in different scallops? What are the functions of the different genes in scallops? Fortunately, sequencing of the scallop genome has greatly facilitated the identification and functional studies of related gene sequences [1, 21]. In the present study, a systematic identification and comprehensive analysis of the TGF-β superfamily was performed in three scallop genomes, including the Bay scallop (*Argopecten irradians*), the Zhikong scallop (*Chlamys farreri*) and the Yesso scallop (*Mizuhopecten yessoensis*). Characteristics of these genes were identified, including sequence structure, phylogenetic relationships, and selection pressure. Using transcriptome resources, we investigated the expression distribution of these genes in different tissues and at different developmental stages, as well as the expression patterns under different stress levels. The results of this study will provide a basis for understanding the gene structure, evolution, and function of the TGF-β superfamily in scallops.

## Results

### Identification and characterization of TGF-β superfamily proteins

Three scallop species have the same number of TGF-β genes, up to 12. The amino acid sequences of the identified TGF-β superfamily genes are given in Supplementary Table S1. The properties of all the identified TGF-β proteins were predicted and listed in Table 1. The AA length varied from 286 to 505. The molecular weight varied from 32.35 to 58.52 kDa, and the theoretical PI value varied from 5.53 to 10.05. The minimum instability index was 38.24, while the maximum value was 66.15. The aliphatic index ranged from 66.72 to 86.65. The maximum and minimum values for the grand average of hydropathicity were − 0.305 and − 0.84, respectively.

**Table 1:** Protein sequence characteristics of the identified TGF-β superfamily genes in scallops

Gene ID	Number of amino acid (AA)	Molecular weight (Da)	Theoretical Isoelecric point (PI)	Instability index	Aliphatic index	Grand average of hydropathicity
AiTGFB-01	407	46892.54	9.44	48.33	75.63	-0.652
AiTGFB-02	500	58295.62	9.99	63.37	69.76	-0.840
AiTGFB-03	424	49309.58	9.74	48.98	71.72	-0.566
AiTGFB-04	433	50082.13	5.93	56.19	85.89	-0.398
AiTGFB-05	504	58507.32	9.36	58.42	71.01	-0.804
AiTGFB-06	328	37816.73	10.04	38.24	78.32	-0.605
AiTGFB-07	459	53375.25	5.53	59.91	72.03	-0.680
AiTGFB-08	406	46331.69	6.13	47.83	86.65	-0.453
AiTGFB-09	492	56935.67	9.00	66.15	69.70	-0.717
AiTGFB-10	286	32348.63	6.00	47.24	79.69	-0.370
AiTGFB-11	395	44580.82	9.95	52.87	80.63	-0.486
AiTGFB-12	320	36004.40	6.17	42.62	80.41	-0.305
CfTGFB-01	406	46973.68	9.40	51.96	77.93	-0.660
CfTGFB-02	503	58460.99	10.02	66.01	72.05	-0.788
CfTGFB-03	418	48557.57	9.47	44.39	69.47	-0.560
CfTGFB-04	428	49566.39	5.99	52.48	86.00	-0.454
CfTGFB-05	505	58521.63	9.46	63.63	74.14	-0.748
CfTGFB-06	364	41436.89	9.79	45.56	84.01	-0.401
CfTGFB-07	421	48344.81	5.89	47.67	83.80	-0.503
CfTGFB-08	457	52852.99	5.61	55.63	74.49	-0.612
CfTGFB-09	378	43784.11	8.83	54.68	86.32	-0.344
CfTGFB-10	473	53834.28	9.35	56.02	66.72	-0.697
CfTGFB-11	399	45499.72	10.04	62.69	79.07	-0.484
CfTGFB-12	350	40252.09	5.63	52.08	77.40	-0.426
MyTGFB-01	406	46826.51	9.55	50.89	77.46	-0.644
MyTGFB-02	503	58516.20	10.05	65.46	72.82	-0.781
MyTGFB-03	421	48804.86	9.62	46.73	67.62	-0.613
MyTGFB-04	431	49880.63	5.68	55.89	83.60	-0.453
MyTGFB-05	505	58477.57	9.44	60.56	75.11	-0.709
MyTGFB-06	366	41887.51	9.85	46.41	79.81	-0.467
MyTGFB-07	410	47058.45	6.87	50.03	82.49	-0.501
MyTGFB-08	457	53064.34	5.74	58.30	76.39	-0.594
MyTGFB-09	487	55410.99	9.00	54.67	70.66	-0.653
MyTGFB-10	397	44952.00	9.94	56.35	78.99	-0.466
MyTGFB-11	379	43933.13	9.04	42.43	79.13	-0.407
MyTGFB-12	351	40199.83	5.90	56.82	73.82	-0.525

### Phylogenetic analysis of TGF-β superfamily genes

**Fig. 1:**
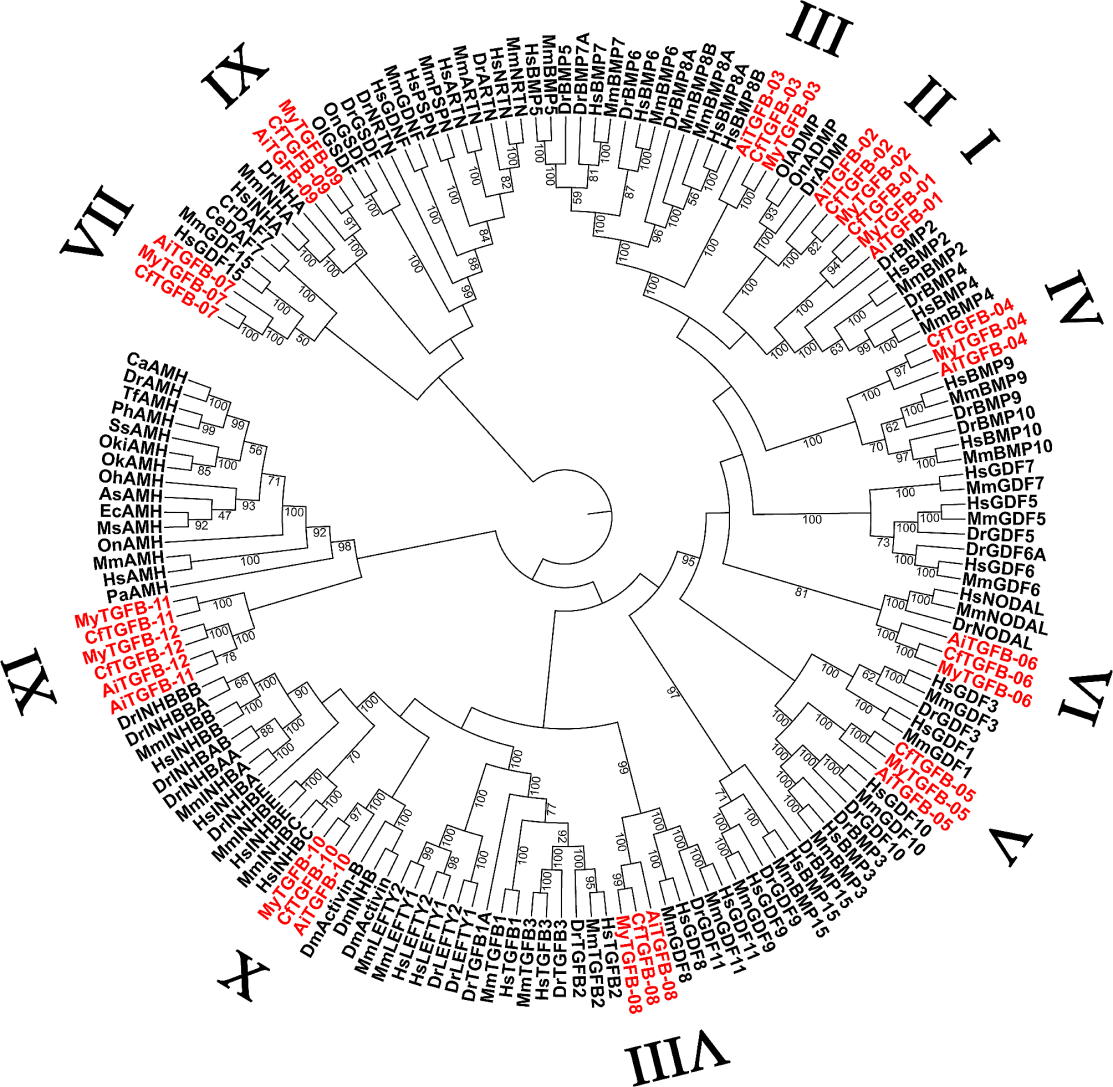
Phylogenetic tree of TGF-β superfamily protein sequences. This tree consists of 164 amino acid sequences of TGF-β superfamily genes from scallops (marked in red) and other representative species. The full names of the species and the corresponding accession numbers of the TGF-β proteins are listed in Supplementary Table S3

Phylogenetic analysis was performed using the TGF-β protein sequences from a variety of animals, including mammals, fishes, insects, and roundworms. As shown in Fig. 1, all scallop TGF-β proteins were clearly grouped into 11 clusters (cluster I to cluster XI). Except for cluster XI, the other 10 clusters contained three members each from three scallop species. Cluster XI contained 6 genes and each species had two genes. The clusters from I to XI showed close phylogenetic relationships with BMP2/4, ADMP, BMP5-8, BMP9/10, BMP3/GDF10, NODAL, GDF15, GDF8/11, INHA, activin/INHB, and AMH, respectively (Table 2).

**Table 2:** Putative cluster of TGF-β superfamily members in three scallop species

Cluster	Gene name		
	*A. irradians*	*C. farreri*	*M. yessoensis*
BMP2/4-like	AiTGFβ-01	CfTGFβ-01	MyTGFβ-01
ADMP-like	AiTGFβ-02	CfTGFβ-02	MyTGFβ-02
BMP5-8-like	AiTGFβ-03	CfTGFβ-03	MyTGFβ-03
BMP9/10-like	AiTGFβ-04	CfTGFβ-04	MyTGFβ-04
BMP3/GDF10-like	AiTGFβ-05	CfTGFβ-05	MyTGFβ-05
NODAL-like	AiTGFβ-06	CfTGFβ-06	MyTGFβ-06
GDF15	AiTGFβ-07	CfTGFβ-07	MyTGFβ-07
GDF8/11-like	AiTGFβ-08	CfTGFβ-08	MyTGFβ-08
INHA-like	AiTGFβ-09	CfTGFβ-09	MyTGFβ-09
Activin/INHB-like	AiTGFβ10	CfTGFβ-10	MyTGFβ10
AMH-like	AiTGFβ-11	CfTGFβ-11	MyTGFβ-11
AMH-like	AiTGFβ-12	CfTGFβ-12	MyTGFβ-12

**Gene structure, motif, and genomic distribution**

**Fig. 2:**
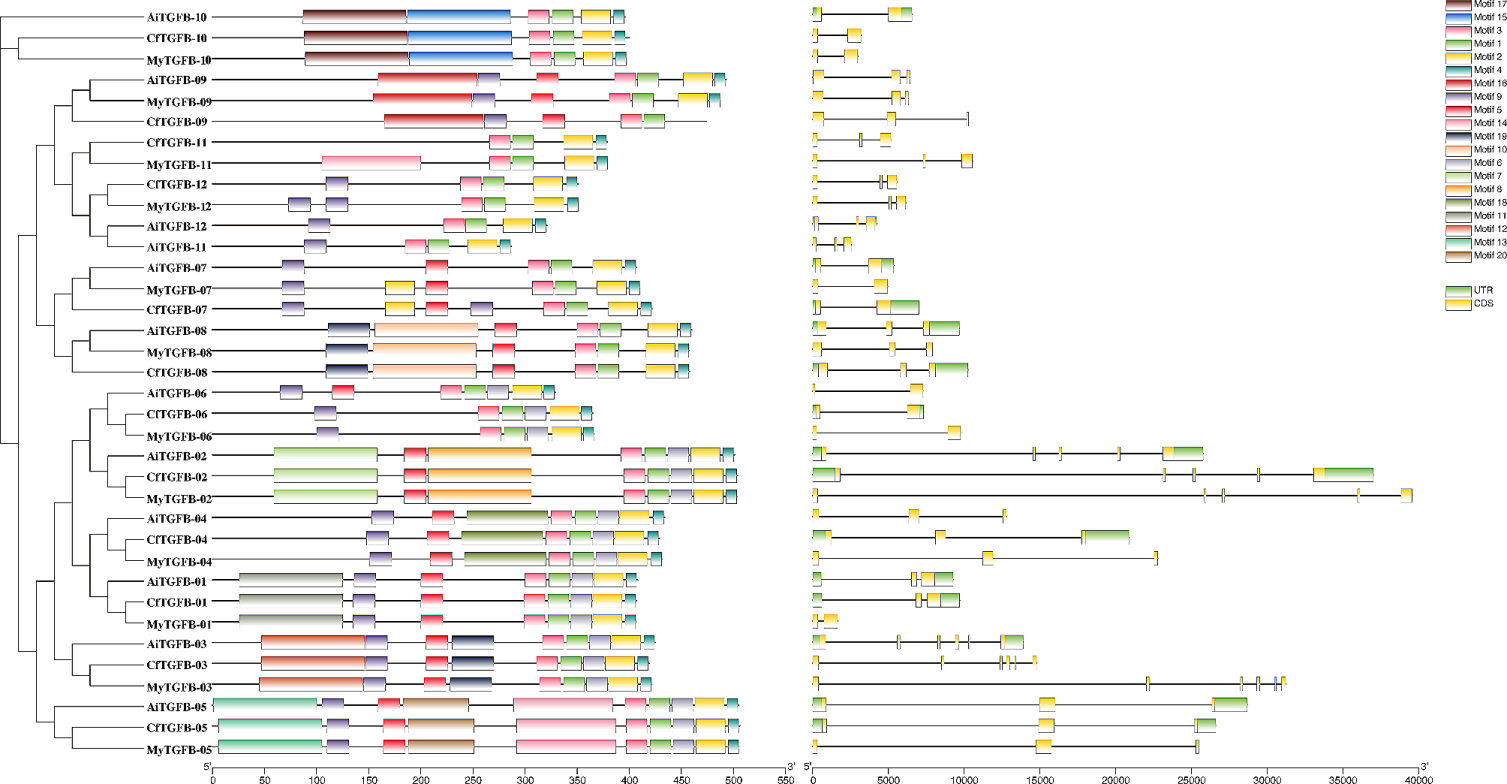
Conserved motif composition and exon-intron structure of TGF-β superfamily members in scallops. The conserved motifs, numbered from 1 to 20, are indicated by different colored boxes

**Fig. 3:**
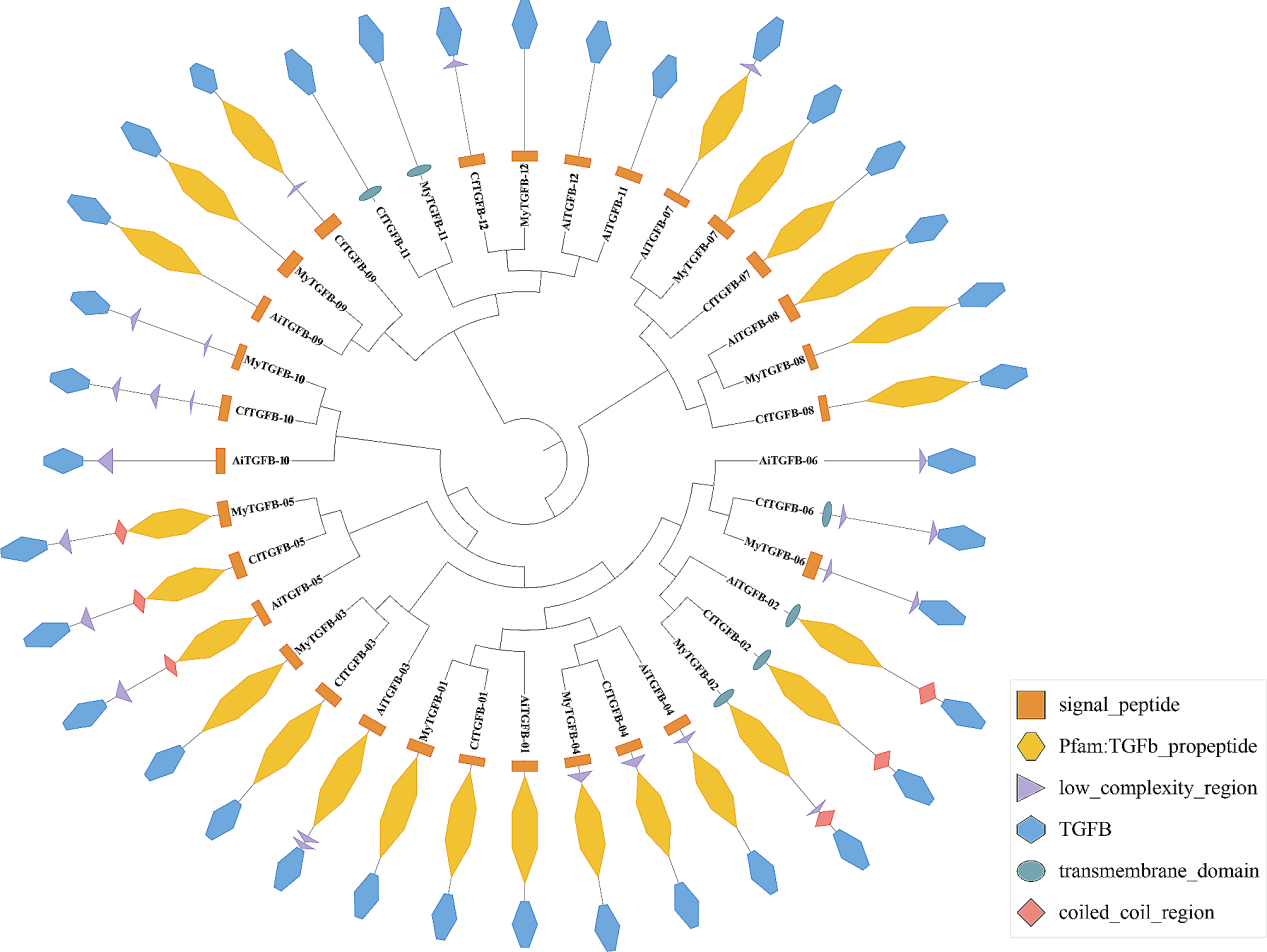
Conserved domains of the TGF-β superfamily proteins in scallops

The conserved motif composition and exon-intron diversification of the TGF-β superfamily genes are shown in Fig. 2. Proteins in the same cluster had more similar motif structural features. Motifs 1–4 were included in the TGF-β domain. Seven conserved cysteine residues were present in these 4 motifs (Table 3). In addition, the number of exons in the TGF-β superfamily genes ranged from 2 to 6. Genes in the same cluster had the same number of exons. For example, the cluster III (BMP5-8-like) genes had 6 exons, while the cluster II (ADMP-like) genes had 5 exons. The other identified genes had 2 or 3 exons. It was suggested that all TGF-β superfamily genes contain the TGF-β domain, most of the identified genes contained a signal peptide and a Pfam: TGF-β propeptide, and several genes contained a low complexity region and a transmembrane domain. Only the ADMP-like and BMP3/GDF10-like genes contained a coiled coil region (Fig. 3).

**Table 3:** Substantially conserved motifs of the TGF-β superfamily in scallops. Seven conserved cysteine residues (C) are underlined in motifs 1–4

MEME Motif	Amino Acid Sequence	Length	Pfam Domain
1	WIIAPKGYEAYYCVGECPFPL	21	TGF-β
2	SVPTPCCVPTKLSPJSLLYFDENENVVLK	29	TGF-β
3	NRESDICCRRPLYVDFHDIGW	21	TGF-β
4	PBMVVKSCGCR	11	TGF-β
5	YAKNAGWETFDVTBAVLKWINS	21	-
6	ESLNPTNHAIIQSLVHAJTPG	21	-
7	FFGIDRVPARVIHKSPPQFMIDLYASITEPGGLVKRESPYRADVIRSFPDREWRQQMHFYYNVSYLSEGEKLLAAEFHIFKLNPRPSSEGEQMPSHVIEI	100	-
8	AVNYGFLVTTTSPSGRHVNGSYVRFAQRKEHHESKQPILVAYTDDGMNRHPSYISPTDENYMQIKKDILKKQRQRMRQFKGKDFRQAIRLLNEREREEDQ	100	-
9	FBVSSITHSEVVTRAEJRJYKD	22	-
10	EKYENAQMQSDSPSRRKEEKLRYQDVQEEYGQPERTYSFAKELPADMSSEFENTIYFDMQVAPEKETNKALLWVYINPDDIIDKNMTEIYVYTIDPPGKF	100	-
11	QNNLFDNKFLDNVDSQQKKEILEAFESSLLNLFSLNARPRPKKDIKIPQYMIDLYKTHTKDPDVLSPNFNIRGKGVGTANTVRSFYHKDAEEHPVQMTGC	100	-
12	GRDKREMQHEILTLLGLHHRPKPAGHSTTDYSAPRFMLNLYNSITSDGGIVDDGNRPQFDRNVTIENEIEPIEGSDVIMSFVNHAKKIKHLRRQRDRTFY	100	-
13	MFHTVNLTVLLGVLVCVVLSREVSDGPQNVLLNESVKTLLGFKDEHPRDQVSSPYGVGQEAPKYMLDLYDRFRNSQISKGHLSGNTVRSIHAEIAEVNGE	100	-
14	SVQRRQKRSIFNNEIPEDPADYDNFHRKFNIPQTHPDILQSRRESRHKISDSRLIPYPDEDRRKNRRNNRKNRKNKRKNRRRKNKNSNLLFPKEWD	96	-
15	PGEVVTTLRADVKKTHWFKLGIPKQLVENAMLSEDQILRLHVHCRGCGRRVQLVLVHGSRRRRKSKGGKRKGSMRVMQPRKRPKRRLSQTRPFLILHTKV	100	-
16	PRAKVNMMKLKEQEHIENIKRRVLEKLRLDAPPKLSGPRPALPFKHLHQEYLSDGVDPGRRRRTLPEFYARKKQVLVMGTDVTTECTNRKSTGCYH	96	-
17	TDPPRGDYYAELLEVISFSEPAESFRDENIIQFKVVRDSQGRKLEVKSANVLVKLRYRRSKKRGRTSCRQKNRRSDVKKKPRRSRCKIIIVLSTVSEDGT	100	-
18	ENVFHSVSYGEMDIDARPKTRTEPLLVVFSSEYSKNKLHMKERHEMVTHEMDSFDFMGDLNDTQTNQSESNTLQHRFKR	79	-
19	RDGLFRKRIFLKKNRDVKPEKLGVLGRPGPEYKRPPKVPYF	41	-
20	DTPHLFALKFQVEWQNGKVKDVALKKFIRHHSMPFLIIYSNDTQNNELDSLENLAEKMHKEKKE	64	-

### Selective pressure analysis

The results of the selection pressure evaluation are shown in Table 4. In the branch model, the ω for the M0 model was 0.130, indicating that the TGF-β superfamily genes were under strong purifying selection. A comparison between M1 and M0 showed that different branches had similar ω values (*P*_LRT_ > 0.05). The site model was then used to identify the positive selection sites. In the site model, the comparison between M3 and M0 suggested that variable alternative pressure existed among different sites (*P*_LRT_ = 0). However, by comparing M2a/M1a and M8/M7, it can be concluded that there were no significant positive selection sites in the identified genes. Overall, the TGF-β superfamily genes in scallops were mainly constrained by purifying selection events.

**Table 4:** Parameter estimates and likelihood value tests for both the branch and site models

Branch Model	Model Type	np	LnL	Estimates of parameters	Model comparison	*P*_LRT_
	0	71	-13405.594	ω = 0.13009	M1/M0	0.1561
	1	72	-13404.588			
Site Model	M0	71	-13315.825	ω = 0.11829	M3/M0	0.0000
	M3	75	-13142.984	p: 0.06077, 0.41136, 0.52786; w: 0.00392, 0.06801, 0.17790		
	M1a	72	-13315.230	p: 0.99524, 0.00476; w: 0.11821, 1.00000	M2a/M1a	1.0000
	M2a	74	-13315.230	p: 0.99524, 0.00130, 0.00346; w: 0.11821, 1.00000, 1.00000		
	M7	72	-13173.316	p = 1.07769, q = 8.56326	M8/M7	0.9984
	M8	74	-13173.318	p0 = 0.99999, p = 1.07769, q = 8.56327, (p1 = 0.00001), w = 1.00000		

### Spatiotemporal expression profiles of TGF-β superfamily genes in scallops

**Fig. 4:**
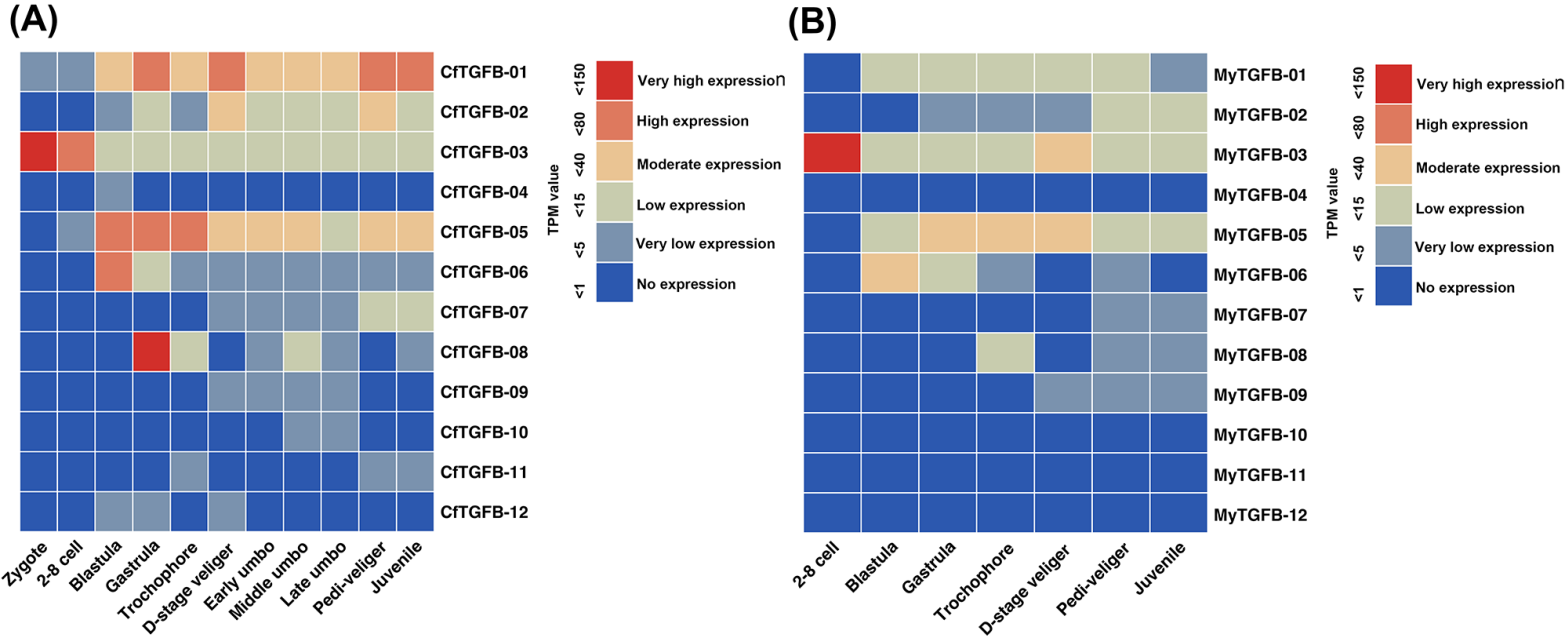
Temporal expression profiles of the TGF-β superfamily genes in the early developmental stages of *C. farreri* (**A**) and *M. yessoensis* (**B**)

Similar expression patterns in the early developmental stages were first analysed in *C. farreri* (Fig. 4, A) and *M. yessoensis* (Fig. 4, B). CfTGFβ-03 (BMP5-8-like) showed high expression at the zygote and 2–8 cell stage, and MyTGFβ-03 (BMP5-8-like) was highly expressed at the 2–8 cell stage. CfTGFβ-06 and MyTGFβ-06, which were NODAL-like proteins, showed high and moderate expression at the blastula stage, respectively. At the gastrula, trochophore and D-stage veliger stage, CfTGFβ-05 and MyTGFβ-05, which were BMP3/GDF10-like proteins, showed high and moderate expression, respectively. Several genes showed species-specific expression patterns in *C. farreri* and *M. yessoensis*. GDF8/11-like (CfTGFβ-08) was highly expressed in the gastrula and was not detected in *M. yessoensis*. CfTGFβ-01 (BMP2/4-like) was highly or moderately expressed from the blastula to juvenile stages, whereas MyTGFβ-01 (BMP2/4-like) was expressed at low levels.

**Fig. 5:**
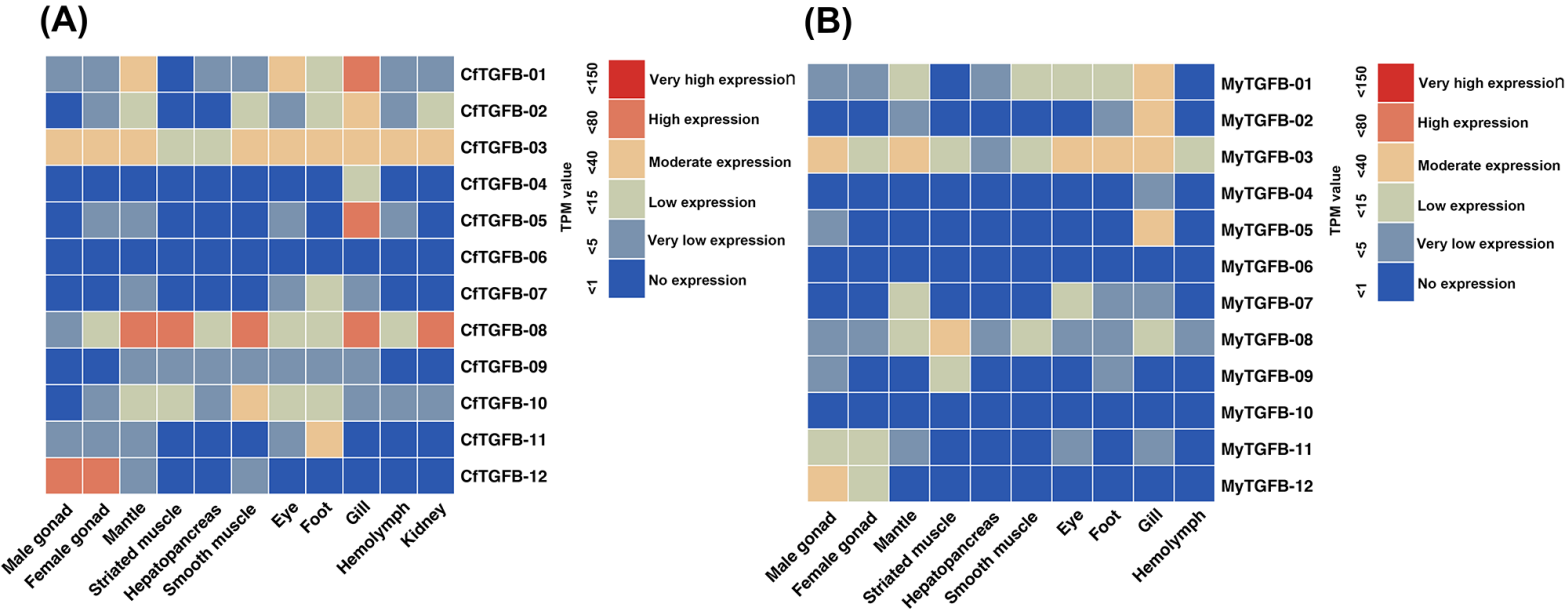
Spatial expression profiles of the TGF-β superfamily genes in the adult tissues of *C. farreri* (**A**) and *M. yessoensis* (**B**)

**Fig. 6:**
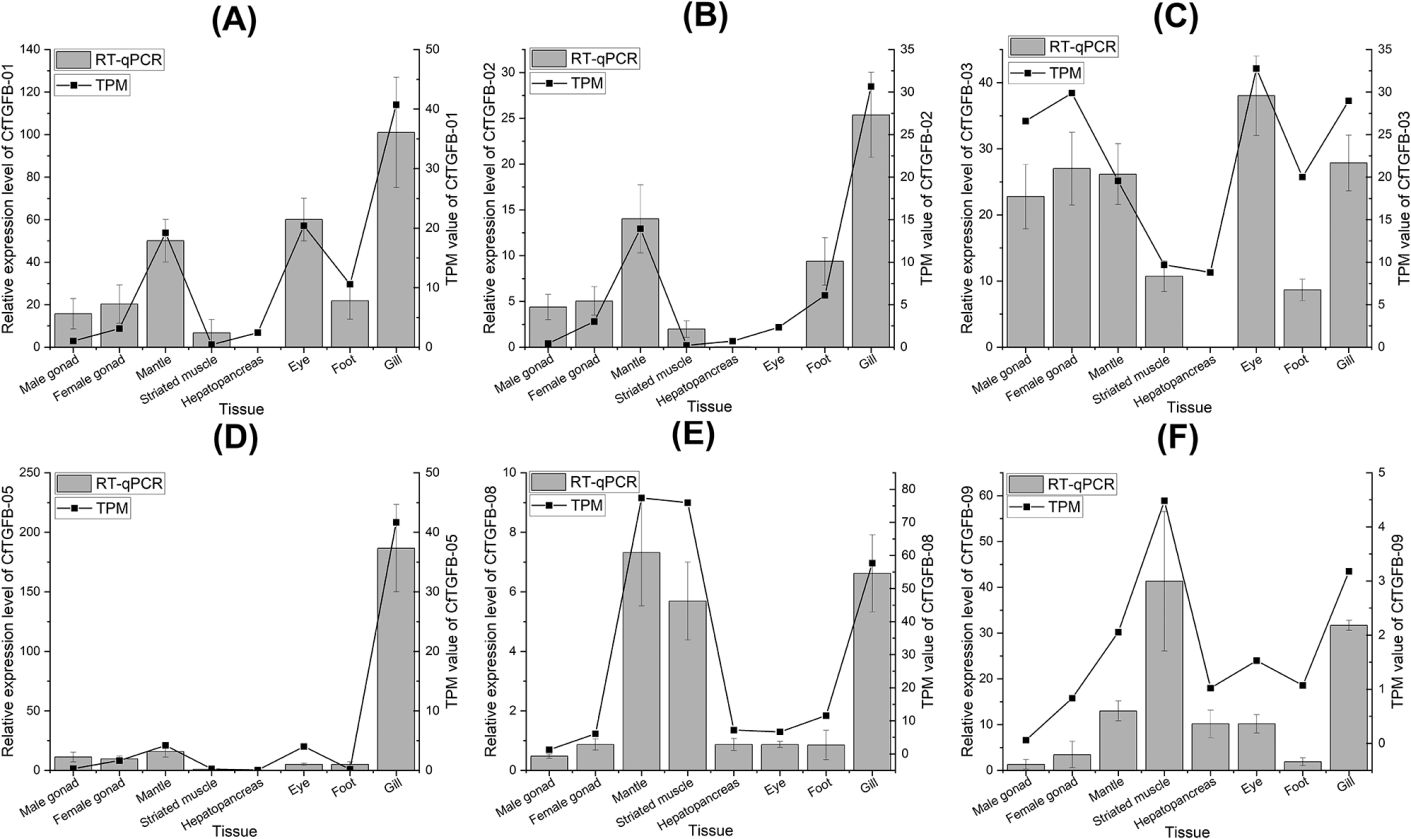
Validation of spatial expression profiles of CfTGFβ-01 (**A**), CfTGFβ-02 (**B**), CfTGFβ-03 (**C**), CfTGFβ-05 (**D**), CfTGFβ-08 (**E**), and CfTGFβ-09 (**F**) in *C. farreri*. These data by RT-qPCR are expressed as the mean ± SD relative to the reference gene. The histogram represents the relative expression detected by RT-qPCR. The line graph represents TPM in the transcriptome

The spatial expression profiles of the TGF-β superfamily genes in adult tissues are shown in Fig. 5. Furthermore, the patterns of the gene expression of six CfTGF-β genes were verified by RT-qPCR, the results of which were consistent with the RNA-Seq analyses (Fig. 6). Interestingly, CfTGFβ-12 (AMH-like) was highly expressed in the gonads. MyTGFβ-12 (AMH-like) also showed specifically moderate expression levels in the male gonad. CfTGFβ-08 (GDF8/11-like) was highly expressed in mantle, striated muscle, smooth muscle, gill, and kidney, while MyTGFβ-08 (GDF8/11-like) showed higher expression in striated muscle than in other adult tissues. CfTGFβ-10 (activin/INHB-like) also showed a moderate expression level in smooth muscle. CfTGFβ-03 (BMP5-8-like) and MyTGFβ-03 (BMP5-8-like) were ubiquitously expressed in adult tissues. CfTGFβ-01, MyTGFβ-01, CfTGFβ-02, MyTGFβ-02, CfTGFβ-05 and MyTGFβ-05 showed high or moderate expression in the gill. In addition, the genes in cluster IV (BMP9/10),VI (NODAL-like), VII (GDF15-like), and IX (INHA-like) showed no or very low expression in the measured adult tissues. The TGF-β superfamily genes were not expressed in the hepatopancreas.

**Fig. 7:**
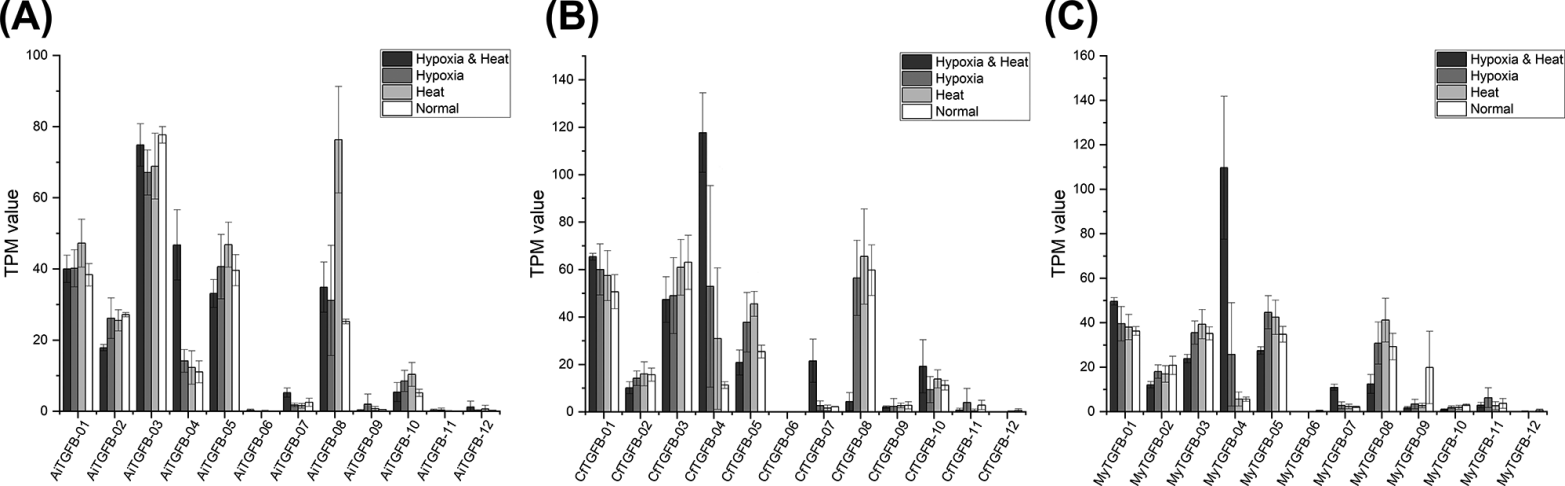
The expression profiles of TGF-β superfamily genes under heat stress, hypoxia stress or heat plus hypoxia stress in *A. irradians* (**A**), *C. farreri* (**B**) and *M. yessoensis* (**C**)

The expression profiles of TGF-β superfamily genes under heat stress or hypoxia stress in *A. irradians*, *C. farreri* and *M. yessoensis* are shown in Fig. 7. The statistical results are shown in Supplementary Table S2. Interestingly, the genes in cluster IV (AiTGFβ-04, CfTGFβ-04, MyTGFβ-04) were highly expressed under heat plus hypoxia stress in the three scallop species. Compared to the normal condition, the gene expression levels of genes in cluster VII (CfTGFβ-07 and MyTGFβ-07) were significantly different under heat plus hypoxia stress, which showed no difference only under heat stress or hypoxia stress alone. In addition to AiTGFβ-04, there was no differentially expressed gene (DEG) under heat and hypoxia stress in *A. irradians*, while there were three DEGs (CfTGFβ-01, CfTGFβ-08, CfTGFβ-11) and two DEGs (MyTGFβ-09, MyTGFβ-10) in *C. farreri* and *M. yessoensis*, respectively.

## Discussion

To date, the number of TGF-β superfamily genes has been extensively studied in various animals, showing considerable differences among different organisms [22, 23]. In this study, a comprehensive survey of the TGF-β superfamily was carried out in scallops. Three scallop species had the same number of members in the TGF-β superfamily, which was much lower than that observed in many vertebrates [4]. For example, there are at least 30 in mammals [4, 24]. The number of TGF-β superfamily members in scallops was higher than that in oysters and some other invertebrates, such as fruit flies, leeches, and jellyfish [4]. The expansion of family members probably originated from the duplication of a common ancestral gene and was widely dispersed by chromosomal translocations [2, 25]. Gene duplication has been proposed as a primary mechanism for increasing organismal complexity and generating evolutionary novelty. There has been evidence for two rounds of genome duplication (2R) in vertebrates and additional rounds (3R or 4R) in teleosts [4, 26].

Previous studies have suggested that the first members of the TGF-β superfamily to appear were BMPs/GDFs, which subsequently differentiated into activin/inhibin, while the TGF-βs and LEFTY were more recent, appearing only in deuterostomes [2, 27, 28]. Consistent with previous findings, the current analysis showed that TGF-βs and LEFTY were absent in scallops. BMP, ADMP, GDF, NODAL, activin/inhibin, and AMH were identified in each scallop genome. The PI from the same cluster showed consistency. Most TGF-β superfamily proteins were unstable in nature (INS > 40). According to the aliphatic index, all proteins showed a hydrophilic nature. The characteristics of the TGF-β superfamily in scallops were similar to those in other species in previous studies [29], which showed that the TGF-β superfamily was evolutionarily conserved. This result can be supported by selection pressure analysis. The TGF-β superfamily genes in scallops have evolved under purifying selection. Different branches showed similar selective pressures, and no site was identified for the positive selection test. In general, purifying selection acts to selectively eliminate deleterious mutations, often resulting in a more conservative gene [30, 31]. In two artificially selective *A. irradians* breeds, the TGF-β type I receptor gene was detected to be selected and no TGF-β superfamily genes were under selection [32]. In general, these results suggested that the TGF-β superfamily was conserved in scallops.

In the current study, TGF-β superfamily genes were specifically expressed at different early developmental stages. BMP5-8-like (CfTGFβ-03 and MyTGFβ-03) were both highly expressed at the 2–8 cell stage. BMP3/GDF10-like (CfTGFβ-05 and MyTGFβ-05) and BMP2/4-like (CfTGFβ-01) showed high expression levels at several developmental stages. BMPs play key roles in gastrulation, mesoderm induction and axial patterning in the embryo [33]. BMP2/4 is a crucial factor for dorsal-ventral patterning in oysters [17]. In jellyfish and leeches, BMP2/4 and BMP5-8 have been implicated in larval axial development [34, 35]. In addition, NODAL-like genes (CfTGFβ-06 and MyTGFβ-06) were specifically highly expressed at the blastula stage. Previous reports have shown that NODAL is needed for early cell fate decisions, organogenesis, left-right development [36, 37], anterior-posterior body axis development [38] and the oral-aboral axis in the embryo [33]. In this study, several BMP-like and NODAL-like genes may play important roles in early development, patterning the embryonic body plan and later regulating development and homeostasis.

Previous studies have shown that GDF8/11 plays a critical role in regulating muscle growth [39]. For example, in *M. yessoensis*, inhibition of myostatin mRNA could increase a combination of hyperplasia and hypertrophy of myosin heavy chain (MHC) II striated myofibers in striated muscle, thereby increasing muscle cellularity [12]. The GDF8 gene is also associated with muscle growth in other scallops [11, 40]. SNPs in the myostatin gene have been developed as candidate molecular markers for selective breeding in *C. farreri* [10, 41] and the Noble scallop (*Chlamys nobilis*) [42]. Similar results were obtained in this study, where the GDF8/11-like gene (CfTGFβ-08) showed high expression in striated muscle and smooth muscle. MyTGFβ-08 also showed moderate expression in striated muscle, with low or no expression in other tissues. The results were consistent with previous studies in *M. yessoensis* [12, 43]. In addition, BMP5-8-like (CfTGFβ-03) and CfTGFβ-10 (activin/INHB-like) showed moderate expression levels in smooth muscle. To date, data on BMP5-8 and activin/inhibin in invertebrates are very limited and have rarely been reported in scallops. Activin/inhibin has been suggested to play an important role in spermatogenesis in mammals [44] and in the regulation of oocyte maturation in fish [45, 46]. Therefore, GDF8/11 can regulate muscle growth in scallops as in other species, and how other TGF-β superfamily genes are involved in muscle development should be further investigated in scallops.

Several TGF-β members have also been identified as sex determination/differentiation genes. BMP2a and BMP10a showed gonad-specific expression in *M. yessoensis* and the expression level of BMP2a showed seasonal changes at different gonad maturational stages [13]. In this study, there were one BMP2/4-like gene and one BMP9/10-like gene in all three scallop species. However, these two genes were not specifically expressed in the gonad in either *C. farreri* or *M. yessoensis*. This difference may be due to the different developmental stages of the gonads. In the current study, AMH-like genes (CfTGFβ-12 and MyTGFβ-12) showed specific expression levels in the gonad. “Amh-amhy-amhr2” acts as a master sex-determining gene in teleost fish, regulating germ cell proliferation and gonad development [47, 48]. Interestingly, AMH is duplicated in some fish, such as amhy (AMH on the Y chromosome) in Nile tilapia [49, 50], amhby (Y chromosome-specific copy of AMH) in northern pike [47], and amhy (Y-linked duplicates of AMH) in Patagonian pejerrey [51] and Sebastes rockfish [52]. In these species, a duplicate copy of AMH acts as a master sex-determining gene [48]. In Nile tilapia, loss of amhy function in XY fish resulted in male to female sex reversal, while overexpression of AMH resulted in female to male sex reversal [53]. *C. farreri* and *M. yessoensis* are gonochoristic species and the ZW-type sex chromosomes are homomorphic chromosomes [54]. AMH is also needed to drive testicular development in a reptilian species, the Chinese soft-shelled turtle, a typical species exhibiting ZZ/ZW sex chromosomes [55]. In scallops, the genes highly expressed in the gonads were from cluster XI. The 6 genes in this cluster shared a high nucleotide identity, and the genes in scallops may have similar functions in other species. Therefore, there may be a duplication of autosomal AMH that was later translocated to the ancestral sex chromosome. This information here provides new insights into the important role of AMH in gonadal growth/maturation in scallops.

The expression profiles of TGF-β superfamily genes under heat stress or hypoxia stress were significantly different from those under heat plus hypoxia stress in scallops. For example, the genes in cluster IV (BMP9/10-like) were both highly expressed under heat plus hypoxia stress in three scallop species. These observations indicated that BMP9/10-like genes may be involved in the combined stress of multiple factors. The genes in cluster VII were significantly differentially expressed under heat stress and hypoxia stress in both *C. farreri* and *M. yessoensis*. TGF-β superfamily genes are known to control a wide range of biological processes, including immunosuppression and apoptosis induction. There is evidence that hypoxia stress can induce apoptosis, inflammation, and autophagy in marine bivalves [56]. TGF-β transcription increased in Nile tilapia [57] and rainbow trout [58] during exposure to hypoxia. BMP-4 was significantly downregulated under short-term salinity stress in abalone [59]. However, few studies have reported the function of the TGF-β superfamily in stress tolerance in scallops. In general, this study provided a fundamental clue for understanding the important roles of the TGF-β superfamily in stress tolerance in scallops.

## Conclusions

The present study is the first report of a comparative genome-wide characterization of the TGF-β superfamily in scallops. All three scallop species had the same number of TGF-β superfamily genes. The phylogenetic tree supported that these genes were grouped into 11 clusters. Selective pressure analysis showed that the scallop TGF-β superfamily has evolved under strong purifying selection. The spatiotemporal expression of TGF-β genes suggested that different TGF-β members have diverse functions in growth and development. Furthermore, the results provide insight into the potential effects of the TGF-β superfamily on gonadal growth/maturation and stress tolerance in scallops. Taken together, our findings provide global insights into the phylogeny and expression patterns of TGF-β superfamily genes, which are multifunctional cytokines capable of regulating a wide range of cellular behaviors in scallops.

## Methods

### TGF-β sequence identification

The genome and annotation files of three scallop species, including *A. irradians*, *C. farreri*, and *P. yessoensis*, were downloaded. The transforming growth factor β-like domain query (accession: PF00019) was first downloaded from the InterPro database (https://www.ebi.ac.uk/interpro/). The HMMER package V3.3.2 was then used to search for TGF-β proteins in each genome. The initial threshold expectation value was set to 1. The non-redundant sequences were analysed for the presence of the PF00019 domain using online SMART analysis [60] with a threshold of 1e-5. The protein sequence characteristics of TGF-β in three scallop species, including amino acid length (AA), molecular weight, isoelectric point (PI), instability index (INS), aliphatic index, and grand average of hydropathicity, were predicted using TBtools software v1.098 [61].

### Phylogenetic analyses

A set of TGF-β protein sequences from 19 different species was obtained from the NCBI databases (Supplementary Table S3). All 164 TGF-β sequences, including the retrieved proteins and those identified from three scallop species, were used to construct the phylogenetic tree. Multiple sequence alignments were first generated using MAFFT v7.158b [62]. Phylogenetic trees were then constructed by using IQ-TREE v2.2.0 with the option -m MFP --bnni -B 4000 -T AUTO [63]. Phylogenetic trees were visualized using the iTOL (interactive tree of life) online tool (https://itol.embl.de/) [64].

### Gene structure and protein domain

To illustrate the exon-intron structure of the TGF-β genes, TBtools software was used to generate the gene structure. The MEME website (http://meme-suite.org/) was used to discover the conserved motif of the scallop TGF-β proteins with the following parameters: maximum length of the conserved motif, 100; minimum length, 6; maximum number, 20, and default values for other parameters. The generated preserved motif files were visualized using the iTOL online tool. In addition, the conserved domains of TGF-β proteins in scallops were analysed using the Batch SMART plug-in in TBtools software.

### Selection pressure assessment

Selective pressure was assessed by using the branch and site model in EasyCodeML V1.0 with the default parameters [65]. The branch models assume that the ratios (ω) of nonsynonymous substitution sites (dN) and synonymous substitution sites (dS) vary among branches. For the branch models, the comparison of two models (one ratio and free ratio) was calculated to test whether ω differs among different branches. The site models assume that the ω ratio varies among sites. In the site models, the specific models (M0, M1a, M2a, M3, M7, and M8) were tested by adjusting the parameters. Among these models, the comparison of M3/M0 was used to detect whether the ω ratio was consistent between different sites, while the comparisons of the M2a/M1a and M8/M7 model pairs test were used for positive selection.

### Expression profiling of TGF-β superfamily genes

To understand the spatiotemporal expression patterns of TGF-β superfamily genes in scallops, publicly available RNA-seq data from two scallops were downloaded from the NCBI SRA database (Supplementary Table S4). Raw RNA sequencing reads were trimmed using the NGStoolkit program with the default parameters [66]. The reference genome was then indexed, and the clean reads were mapped to the reference genome using HISAT2 [67]. After the resulting SAM files were converted to BAM files and sorted using SAMtools [68], the transcripts per kilobase per million mapped reads (TPM) value of each gene was determined using StringTie v2.1.7 [69]. TPM values < 1, <5, < 15, <40, < 80 and < 150 were classified as no expression, low expression, moderate expression, high expression, and very high expression, respectively. Heatmaps of the gene expression levels were generated by using the ggplot2 package in R software [70]. In addition, to determine whether TGF-β superfamily genes were involved in environmental stress, RNA-seq data for three scallop species under heat, hypoxia and heat plus hypoxia stress were downloaded from the NCBI SRA database (Supplementary Table S4). Raw transcriptome sequencing files were processed using the same method as described above. In addition, the read count matrix was generated by python script “prepDE.py”, and the significance test of difference analysis was performed using DESeq2 1.42.0 [71]. P values were adjusted using Benjamini and Hochberg’s approach for controlling the false discovery rate (FDR). Genes with padj ≤ 0.05 and |log_2_(fold change)|>1 were considered DEGs. All steps were performed on a desktop computer in a WSL2 environment (Ubuntu22.04) with 12 cores, 64 GB RAM, and 5 TB hard-disk.

### Application of quantitative real-time PCR for expression profile validation

To assess the transcriptome sequencing findings by RT-qPCR, CfTGFβ-01, CfTGFβ-02, CfTGFβ-03, CfTGFβ-05, CfTGFβ-08, and CfTGFβ-09, were selected randomly. The male gonad, female gonad, mantle, striated muscle, eye, foot, hepatopancreas and gill were collected from 9 healthy *C. farreri* scallops, and three individuals were put together as one sample. TRIzol reagent (Gibco BRL, USA) was used to extract total RNA from tissues. cDNA was synthesized using the PrimeScript™ RT reagent Kit with gDNA Eraser kit (Takara, Japan). The RT-qPCR reactions were carried out using SYBR (TOYOBO, Osaka, Japan). The gene-specific primers were designed using Primer 5.0 (Supplementary Table S5), and actin was used as the reference gene [72]. There were four technical duplicates of each sample during RT-qPCR. Finally, the relative expression level was calculated with the 2^−ΔΔCT^ method, and the RT-qPCR results were compared with the transcriptome data.

### Electronic supplementary material

Below is the link to the electronic supplementary material.

Supplementary Material 1

## Data Availability

The datasets generated and/or analyzed during the current study are available in the NCBI repository [PRJNA259405, PRJNA428789, PRJNA185465, PRJNA259405, and PRJNA786240], [PERSISTENT WEB LINK OR ACCESSION NUMBER TO DATASETS], cfbase [http://mgb.ouc.edu.cn/cfbase/html/].

## References

[1] Li Y, Sun X, Hu X, Xun X, Zhang J, Guo X, Jiao W, Zhang L, Liu W, Wang J (2017). Scallop genome reveals molecular adaptations to semi-sessile life and neurotoxins. Nat Commun.

[2] Liu S, Guo J, Cheng X, Li W, Lyu S, Chen X, Li Q, Wang H (2022). Molecular evolution of transforming growth factor-β (TGF-β) gene family and the functional characterization of lamprey TGF-β2. Front Immunol.

[3] Weiss A, Attisano L (2013). The TGFbeta superfamily signaling pathway. WIREs Dev Biol.

[4] Zheng S, Long J, Liu Z, Tao W, Wang D (2018). Identification and evolution of TGF-β signaling pathway members in twenty-four animal species and expression in Tilapia. Int J Mol Sci.

[5] Chang H, Brown CW, Matzuk MM (2002). Genetic analysis of the mammalian transforming growth factor-β superfamily. Endocr Rev.

[6] Pan Q, Kay T, Depince A, Adolfi M, Schartl M, Guiguen Y, Herpin A (2021). Evolution of master sex determiners: TGF-β signalling pathways at regulatory crossroads. Phil Trans R Soc B.

[7] Wu MY, Hill CS (2009). TGF-β superfamily signaling in embryonic development and homeostasis. Dev Cell.

[8] Tzavlaki K, Moustakas A, TGF-β Signaling (2020). Biomolecules.

[9] Sawatari E, Seki R, Adachi T, Hashimoto H, Uji S, Wakamatsu Y, Nakata T, Kinoshita M (2010). Overexpression of the dominant-negative form of myostatin results in doubling of muscle-fiber number in transgenic medaka (*Oryzias latipes*). Comp Biochem Physiol A.

[10] Fu Q, Guo H, Feng L, Li X, Zhang L, Wang S, Hu X, Bao Z (2016). Association of *myostatin* variants with growth traits of Zhikong scallop (*Chlamys Farreri*). J Ocean Univ China.

[11] Kim HW, Mykles DL, Goetz FW, Roberts SB (2004). Characterization of a myostatin-like gene from the bay scallop, *Argopecten irradians*. Biochim Biophys Acta.

[12] Sun X, Li L, Liu Z, Zhao D, Yang A, Zhou L, Wu B, Tian J (2020). Molecular characterization of the myostatin gene and its regulation on muscle growth in Yesso scallop *Patinopecten Yessoensis*. Aquaculture.

[13] Konuma M, Nagasawa K, Mokrina M, Kobayashi M, Osada M (2021). Gonadal somatic cell-specific transforming growth factor-β superfamily member in the Yesso scallop reveals gonadal somatic cell distribution during the reproductive phase. Gene.

[14] Corporeau C, Groisillier A, Jeudy A, Barbeyron T, Fleury E, Fabioux C, Czjzek M, Huvet A (2011). A functional study of transforming growth factor-beta from the gonad of Pacific oyster *Crassostrea gigas*. Mar Biotechnol.

[15] Chen M, Chen Y, Cao W, Wang C, Ning J, Liu B, Lu X, Wang C (2022). Transcriptomic analyses of hermaphroditic gonads at different stages revealing candidate genes for sex differentiation and gonad growth/maturation in QN Orange scallops. Aquac Res.

[16] Kaneko H, Ijiri S, Kobayashi T, Izumi H, Kuramochi Y, Wang DS, Mizuno S, Nagahama Y (2015). Gonadal soma-derived factor (gsdf), a TGF-beta superfamily gene, induces testis differentiation in the teleost fish *Oreochromis niloticus*. Mol Cell Endocrinol.

[17] Tan S, Huan P, Liu B (2017). Expression patterns indicate that BMP2/4 and Chordin, not BMP5-8 and Gremlin, mediate dorsal-ventral patterning in the mollusk *Crassostrea gigas*. Dev Genes Evol.

[18] Tan S, Huan P, Liu B (2022). Molluskan dorsal-ventral patterning relying on BMP2/4 and chordin provides insights into spiralian development and evolution. Mol Biol Evol.

[19] Lelong C, Badariotti F, Le Quere H, Rodet F, Dubos MP, Favrel P (2007). Cg-TGF-β, a TGF-β/activin homologue in the Pacific Oyster *Crassostrea gigas*, is involved in immunity against Gram-negative microbial Infection. Dev Comp Immunol.

[20] Fujio K, Komai T, Inoue M, Morita K, Okamura T, Yamamoto K (2016). Revisiting the regulatory roles of the TGF-β family of cytokines. Autoimmun Rev.

[21] Wang S, Zhang J, Jiao W, Li J, Xun X, Sun Y, Guo X, Huan P, Dong B, Zhang L (2017). Scallop genome provides insights into evolution of bilaterian karyotype and development. Nat Ecol Evol.

[22] Ishimaru Y, Tomonari S, Matsuoka Y, Watanabe T, Miyawaki K, Bando T, Tomioka K, Ohuchi H, Noji S, Mito T (2016). TGF-β signaling in insects regulates metamorphosis via juvenile hormone biosynthesis. Proc Natl Acad Sci USA.

[23] Savage-Dunn C, Padgett RW (2017). The TGF-β family in *Caenorhabditis elegans*. CSH Perspect Biol.

[24] Li S, Wu J (2020). TGF-β/SMAD signaling regulation of mesenchymal stem cells in adipocyte commitment. Stem Cell Res Ther.

[25] Xu S, Zhang S, Zhang W, Liu H, Wang M, Zhong L, Bian W, Chen X (2022). Genome-wide identification, phylogeny, and expression profile of the dmrt (doublesex and mab-3 related transcription factor) gene family in Channel catfish (*Ictalurus punctatus*). Front Genet.

[26] Berthelot C, Brunet F, Chalopin D, Juanchich A, Bernard M, Noël B, Bento P, Da Silva C, Labadie K, Alberti A (2014). The rainbow trout genome provides novel insights into evolution after whole-genome duplication in vertebrates. Nat Commun.

[27] Hinck AP, Mueller TD, Springer TA (2016). Structural biology and evolution of the TGF-β family. CSH Perspect Biol.

[28] Grande C, Martín-Durán JM, Kenny NJ, Truchado-García M, Hejnol A (2014). Evolution, divergence and loss of the nodal signalling pathway: new data and a synthesis across the Bilateria. Int J Dev Biol.

[29] Rehman MS, Hassan FU, Rehman ZU, Ishtiaq I, Rehman SU, Liu Q (2022). Molecular characterization of TGF-beta gene family in buffalo to identify gene duplication and functional mutations. Genes.

[30] Hughes AL, Packer B, Welch R, Bergen AW, Chanock SJ, Yeager M (2003). Widespread purifying selection at polymorphic sites in human protein-coding loci. Proc Natl Acad Sci USA.

[31] Yang Z, Wang X, Gu S, Hu Z, Xu H, Xu C (2008). Comparative study of SBP-box gene family in *Arabidopsis* and rice. Gene.

[32] Wang H, Lv J, Zeng Q, Liu Y, Xing Q, Wang S, Hu J, Bao L (2021). Genetic differentiation and selection signatures in two bay scallop (*Argopecten irradians*) breeds revealed by whole-genome resequencing analysis. Aquaculture.

[33] Duboc V, R$$ \ddot{\text{o}}$$ttinger E, Besnardeau L, Lepage T, Nodal. BMP2/4 signaling organizes the oral-aboral axis of the sea urchin embryo. Dev Cell. 2004;6:397–410. 10.1016/s1534-5807(04)00056-5 15030762

[34] Kuo DH, Weisblat DA (2011). A new molecular logic for BMP-mediated dorsoventral patterning in the leech *Helobdella*. Curr Biol.

[35] Reber-M$$ \ddot{\text{u}}$$ller S, Streitwolf-Engel R, Yanze N, Schmid V, Stierwald M, Erb M, Seipel K. BMP2/4 and BMP5-8 in jellyfish development and transdifferentiation. Int J Dev Biol. 2006;50(4):377–84. 10.1387/ijdb.052085sr 16525932

[36] Grande C, Patel NH (2009). Nodal signalling is involved in left-right asymmetry in snails. Nature.

[37] Yost HJ (1998). Left right development in *Xenopus* and zebrafish. Semin Cell Dev Biol.

[38] Zhou X, Sasaki H, Lowe L, Hogant BLM, Kuehn MR (1993). Nodal is a novel TGF-β-like gene expressed in the mouse node during gastrulation. Nature.

[39] Guo H, Bao Z, Li J, Lian S, Wang S, He Y, Fu X, Zhang L, Hu X (2012). Molecular characterization of TGF-β type I receptor gene (Tgfbr1) in *Chlamys farreri*, and the association of allelic variants with growth traits. PLoS ONE.

[40] Morelos RM, Ramírez JL, García-Gasca A, Ibarra AM (2015). Expression of the myostatin gene in the adductor muscle of the Pacific lion‐paw scallop *Nodipecten subnodosus* in association with growth and environmental conditions. J Exp Zool.

[41] Wang X, Meng X, Song B, Qiu X, Liu H (2010). SNPs in the myostatin gene of the mollusk *Chlamys farreri*: association with growth traits. Comp Biochem Physiol B.

[42] Fan S, Xu Y, Liu B, He W, Zhang B, Su J, Yu D (2017). Molecular characterization and expression analysis of the myostatin gene and its association with growth traits in noble scallop (*Chlamys Nobilis*). Comp Biochem Physiol B.

[43] Kim HW, Kim HJ, Yoo MS (2007). Characterization of a myostatin-like gene from the scallop. Patinopecten Yessoensis.

[44] Cai K, Hua G, Ahmad S, Liang A, Han L, Wu C, Yang F, Yang L (2011). Action mechanism of inhibin α-subunit on the development of sertoli cells and first wave of spermatogenesis in mice. PLoS ONE.

[45] Tan Q, Balofsky A, Weisz K, Peng C (2009). Role of activin, transforming growth factor-β and bone morphogenetic protein 15 in regulating zebrafish oocyte maturation. Comp Biochem Physiol A.

[46] Tan Q, Zagrodny A, Bernaudo S, Peng C (2009). Regulation of membrane progestin receptors in the zebrafish ovary by gonadotropin, activin, TGF-β and BMP-15. Mol Cell Endocrinol.

[47] Pan Q, Feron R, Yano A, Guyomard R, Jouanno E, Vigouroux E, Wen M, Busnel JM, Bobe J, Concordet JP (2019). Identification of the master sex determining gene in Northern pike (*Esox lucius*) reveals restricted sex chromosome differentiation. PLoS Genet.

[48] Oliveira MAD, Filho ASS, Araújo FE. TGF-β superfamily: an overview of amh signaling into sex determination and differentiation in fish. 2023; 66: e23220371.

[49] Eshel O, Shirak A, Lior D, Band M, Zak T, Markovich-Gordon M, Chalifa-Caspi V, Feldmesser E, Weller JI, Seroussi E et al. Identification of male-specific amh duplication, sexually differentially expressed genes and microRNAs at early embryonic development of Nile tilapia (*Oreochromis niloticus*). 2014; 15: 774. 10.1186/1471-2164-15-774 PMC4176596 25199625

[50] Liu X, Dai S, Wu J, Wei X, Zhou X, Chen M, Tan D, Pu D, Li M, Wang D. Roles of anti-M$$ \ddot{\text{u}}$$llerian hormone and its duplicates in sex determination and germ cell proliferation of Nile tilapia. Genetics. 2022;220(3):iyab237. 10.1093/genetics/iyab237 PMC9208641 35100374

[51] Hattori RS, Murai Y, Oura M, Masuda S, Majhi SK, Sakamoto T, Fernandino JI, Somoza GM, Yokota M, Str$$ \ddot{\text{u}}$$ssmann CA. A Y-linked anti-M$$ \ddot{\text{u}}$$llerian hormone duplication takes over a critical role in sex determination. Proc Natl Acad Sci U S A. 2012;109(8):2955–9. 10.1073/pnas.1018392109 PMC3286941 22323585

[52] Song W, Xie Y, Sun M, Li X, Fitzpatrick CK, Vaux F, O’Malley KG, Zhang Q, Qi J, He Y (2021). A duplicated *amh* is the master sex-determining gene for Sebastes rockfish in the Northwest Pacific. Open Biol.

[53] Li M, Sun Y, Zhao J, Shi H, Zeng S, Ye K, Jiang D, Zhou L, Sun L, Tao W, et al. A tandem duplicate of anti-M$$ \ddot{\text{u}}$$llerian hormone with a missense SNP on the Y chromosome is essential for male sex determination in Nile tilapia, *Oreochromis niloticus*. PLoS Genet. 2015;11:e1005678. 10.1371/journal.pgen.1005678 PMC4654491 26588702

[54] Han W, Liu L, Wang J, Wei H, Li Y, Zhang L, Guo Z, Li Y, Liu T, Zeng Q (2022). Ancient homomorphy of molluscan sex chromosomes sustained by reversible sex-biased genes and sex determiner translocation. Nat Ecol Evol.

[55] Zhou Y, Sun W, Cai H, Bao H, Zhang Y, Qian G, Ge C. The role of anti-M$$ \ddot{\text{u}}$$llerian hormone in testis differentiation reveals the significance of the TGF-β pathway in reptilian sex determination. Genetics. 2019;213(4):1317–27. 10.1534/genetics.119.302527 PMC6893390 31645361

[56] Falfushynska H, Piontkivska H, Sokolova IM (2020). Effects of intermittent hypoxia on cell survival and inflammatory responses in the intertidal marine bivalves *Mytilus edulis* and *Crassostrea gigas*. J Exp Biol.

[57] Choi K, Lehmann DW, Harms CA, Law JM (2007). Acute hypoxia-reperfusion triggers immunocompromise in Nile tilapia. J Aquat Anim Health.

[58] Aksakal E, Ekinci D (2021). Effects of hypoxia and hyperoxia on growth parameters and transcription levels of growth, immune system and stress related genes in rainbow trout. Comp Biochem Physiol A.

[59] Boamah GA, Huang Z, Shen Y, Lu Y, Wang Z, Su Y, Xu C, Luo X, Ke C, You W (2022). Transcriptome analysis reveals fluid shear stress (FSS) and Atherosclerosis pathway as a candidate molecular mechanism of short-term low salinity stress tolerance in abalone. BMC Genomics.

[60] Schultz J, Copley RR, Doerks T, Ponting CP, Bork P (2000). SMART: a web-based tool for the study of genetically mobile domains. Nucleic Acids Res.

[61] Chen C, Chen H, Zhang Y, Thomas HR, Frank MH, He Y, Xia R (2020). TBtools: an integrative toolkit developed for interactive analyses of big biological data. Mol Plant.

[62] Katoh K, Standley DM (2013). MAFFT multiple sequence alignment software version 7: improvements in performance and usability. Mol Biol Evol.

[63] Nguyen LT, Schmidt HA, Von Haeseler A, Minh BQ (2015). IQ-TREE: a fast and effective stochastic algorithm for estimating maximum-likelihood phylogenies. Mol Biol Evol.

[64] Letunic I, Bork P (2019). Interactive tree of life (iTOL) v4: recent updates and new developments. Nucleic Acids Res.

[65] Gao F, Chen C, Arab DA, Du Z, He Y, Ho SYW (2019). EasyCodeML: a visual tool for analysis of selection using CodeML. Ecol Evol.

[66] Patel RK, Jain M (2012). NGS QC Toolkit: a toolkit for quality control of next generation sequencing data. PLoS ONE.

[67] Kim D, Langmead B, Salzberg SL (2015). HISAT: a fast spliced aligner with low memory requirements. Nat Methods.

[68] Li H, Handsaker B, Wysoker A, Fennell T, Ruan J, Homer N, Marth G, Abecasis G, Durbin R (2009). The sequence alignment/map format and SAMtools. Bioinformatics.

[69] Pertea M, Pertea GM, Antonescu CM, Chang TC, Mendell JT, Salzberg SL (2015). StringTie enables. Improved reconstruction of a transcriptome from RNA-seq reads. Nat Biotechnol.

[70] R Core Team. R: A language and environment for statistical computing. R Foundation for Statistical Computing, Vienna, Austria. URL. 2018.

[71] Love MI, Huber W, Anders S (2014). Moderated estimation of Fold change and dispersion for RNA-seq data with DESeq2. Genome Biol.

[72] Fu Q, Guo H, Feng L, Li X, Zhang L, Wang S, Hu X, Bao Z (2016). Association of myostatin variants with growth traits of Zhikong scallop (*Chlamys Farreri*). J Ocean Univ China.

